# Non-Engagement Isn’t Always About Motivation: Understanding Barriers in Outpatient Addiction Care

**DOI:** 10.1192/bjo.2026.11534

**Published:** 2026-06-30

**Authors:** Mohammad Ali

**Affiliations:** Addictions Recovery Community (ARC-MK), Milton Keynes, United Kingdom

## Abstract

**Aims:**

Non-engagement in outpatient addiction services is frequently attributed to poor motivation or readiness to change. This evaluation aimed to examine demographic and service-level factors associated with non-attendance, and to test whether documented motivation predicted engagement.

**Methods:**

A retrospective review of all new referrals to a UK outpatient addiction service over 12 months was undertaken (n=214). Demographic, clinical, and appointment data were analysed, including housing status, referral source, clinician continuity, and attendance. Patient feedback recorded during routine contacts was thematically reviewed.

**Results:**

Mean age was 41 years; 62% were male. Primary substances were alcohol (48%), opiates (27%), stimulants (15%), and polysubstance use (10%). Overall DNA rate was 29%, rising to 38% following initial assessment. Housing instability was associated with higher DNA rates (43% vs 23%). Clinician changes were associated with increased non-attendance (DNA 41% vs 24%). No association was found between documented motivation and attendance. Over half of those who initially disengaged (54%) re-presented within 6 months.

**Conclusion:**

Non-engagement in outpatient addiction services is better understood as a dynamic interaction between patient circumstances and service design rather than a simple reflection of motivation. Interventions focused on continuity, flexible scheduling, and proactive follow-up may reduce disengagement more effectively than motivational approaches alone. Reframing non-engagement as a service-level challenge has importantimplications for engagement strategies, clinician attitudes, and outcome evaluation in addiction psychiatry.

